# Non-probabilistic Solution of Uncertain Vibration Equation of Large Membranes Using Adomian Decomposition Method

**DOI:** 10.1155/2014/308205

**Published:** 2014-02-24

**Authors:** Smita Tapaswini, S. Chakraverty

**Affiliations:** Department of Mathematics, National Institute of Technology, Rourkela, Odisha 769 008, India

## Abstract

This paper proposes a new technique based on double parametric form of fuzzy numbers to handle the uncertain vibration equation for very large membrane for different particular cases. Uncertainties present in the initial condition and the wave velocity of free vibration are modelled through Gaussian convex normalised fuzzy set. Using the single parametric form of fuzzy number, the original fuzzy vibration equation is converted first to an interval fuzzy vibration equation. Next this equation is transformed to crisp form by applying double parametric form of fuzzy numbers. Finally the same governing equation is solved by Adomian decomposition method (ADM) symbolically to obtain the uncertain bounds. The present methods are very simple and effective. Obtained results are depicted in terms of plots to show the efficiency and powerfulness of the present analysis. Results obtained by the methods with new techniques are compared with existing results in special cases.

## 1. Introduction

Vibration analysis of large membranes has a great importance in many areas of science and engineering problems. In music and acoustics, membranes constitute major components. In addition, membranes constitute components of microphones, speakers, and other devices. Moreover, membranes may be used to study two-dimensional wave mechanics and propagation. In bioengineering, many human tissues are considered as membranes. The vibration characteristics of an eardrum are important in understanding hearing. The designs of hearing aid devices involve understanding of vibration behaviour of membranes.

In particular vibration equation of very large membranes has been analysed by the authors [1, 2] due to the great importance in many areas of science and engineering. Yildirim et al. [1] obtained the solution of the vibration equation of a large membrane using homotopy perturbation method and Mohyud-Din and Yildirim [2] have studied and analysed the vibration equation of large membrane of fractional order problem.

In general the parameters and initial condition involved in the vibration equation of large membrane are considered as crisp or defined exactly. But in actual practice, rather than the particular value, only uncertain or vague estimates about the variables and parameters are known because those are found in general by some observation, experiment, or experience. So, to handle these uncertainties and vagueness, one may use fuzzy parameters and variables in the governing differential equations. This represents a natural way to model physical systems under uncertainty. Since, it is too difficult to obtain the exact solution of fuzzy differential equations, so one may need a reliable and efficient numerical technique for the solution of fuzzy differential equations. The concept of fuzzy derivative was first introduced by Chang and Zadeh [3], where they proposed the concept of a fuzzy derivative. Dubois and Prade [4] defined and used the extension principle in their approach. The fuzzy differential equations and fuzzy initial value problems are studied by Kaleva [5, 6] and Seikkala [7]. Various numerical methods for solving fuzzy differential equations and fuzzy fractional differential equations are also introduced in [8–29].

The above literature review reveals that the fuzzy differential equations related to the physical systems are always converted to two crisp differential equations to obtain the solution. But, here in the proposed methodology, the fuzzy vibration equation has been converted to a single crisp differential equation using a new concept of double parametric form of fuzzy numbers. Finally the corresponding differential equation is solved by ADM to obtain the fuzzy solution in double parametric form.

Recently, ADM is found to be a powerful tool for the analysis of linear and nonlinear physical problems. In the beginning of 1980, ADM was developed by Adomian [30, 31] and Ismail et al. [32] used to solve Burger's-Huxley and Burger's-Fisher equations. More applications of ADM are cited in [33–36]. The convergence of the ADM is discussed in [37–39].

Our aim in this paper is to apply the ADM [30, 31] for solving the fuzzy vibration equation for large membranes

(1) $$\begin{array}{c} \frac{\partial^{2}\tilde{v}}{\partial r^{2}}+\frac{1}{r}\frac{\partial \tilde{v}}{\partial r}=\frac{1}{{\tilde{c}}^{2}}\frac{\partial^{2}\tilde{v}}{\partial t^{2}},\;r\geq 0,\;\;t\geq 0, \end{array}$$

with fuzzy initial conditions

(2) v~(r,0)=(0.8,1,1.2)f(r),v~′(r,0)=c~g(r),

where $\tilde{v}(r,t)$ represents the uncertain displacement and $\tilde{c}$ is the uncertain wave velocity of free vibration.

Present paper is organized as follows. In Section 2, we give some basic preliminaries related to the present investigation. ADM is applied with the proposed technique in Section 3 for general solution of fuzzy vibration equation for large membrane. In Section 4 uncertain displacement for different time, for radii of the membrane, and also for uncertain wave velocities of free vibration using the fuzzy initial conditions is obtained. Next numerical results and discussions are presented. Finally in the last section conclusions are drawn.

## 2. Preliminaries

In this section, we present some notations, definitions, and preliminaries which are used further in this paper [40–43].

**Definition 1 (fuzzy number)**

A fuzzy number $\tilde{U}$ is convex normalised fuzzy set $\tilde{U}$ of the real line *R* such that

(3) {μU~(x):R⟶[0,1],  ∀x∈R},

where $\mu_{\tilde{U}}$ is called the membership function of the fuzzy set and it is piecewise continuous.

**Definition 2 (Gaussian fuzzy number)**

Let one now define an arbitrary asymmetrical Gaussian fuzzy number, *U* = (*δ*, *σ*_*l*_, *σ*_*r*_). The membership function $\mu_{\tilde{U}}$ of $\tilde{U}$ will be as follows:

(4) μU(x)={exp⁡[−(x−δ)22σl2]for  x≤δexp⁡[−(x−δ)22σr2]for  x≥δ ∀x∈R,

where the modal value is denoted as *δ* and *σ*_*l*_, *σ*_*r*_ denote the left-hand and right-hand spreads (fuzziness) corresponding to the Gaussian distribution. For symmetric Gaussian fuzzy number the left-hand and right-hand spreads are equal; that is, *σ*_*l*_ = *σ*_*r*_ = *σ*. So the symmetric Gaussian fuzzy number may be written as *U* = (*δ*, *σ*, *σ*) and corresponding membership function may be defined as $\mu_{\tilde{U}}(x)=\exp\{-\beta {(}^{x-\delta )2}\}\;\;\forall x\in R$, where *β* = 1/2*σ* ^2^.

**Definition 3 (single parametric form of fuzzy numbers)**

The symmetric Gaussian fuzzy number in single parametric form can be represented as

(5) U~=[u_(α),u−(α)]=[δ−−(logeα)β,δ+−(logeα)β],

where *α* ∈ [0,1].

It may be noted that the lower and upper bounds of the fuzzy numbers satisfy the following requirements:

- $\underline{u}(\alpha )$ is a bounded left continuous nondecreasing function over [0,1],
- $\bar{u}\left(\alpha \right)$ is a bounded right continuous nonincreasing function over [0,1],
- $\;\underline{u}(\alpha )\leq \bar{u}(\alpha )$, 0 ≤ *α* ≤ 1.

**Definition 4 (double parametric form of fuzzy number)**

Using the single parametric form as discussed in Definition 3 one has $\tilde{U}=[\underline{u}(\alpha ),\overset{-}{u}(\alpha )]$. Now one may write this as crisp number with double parametric form as $\tilde{U}(\alpha ,\beta )=\beta \left(\overset{-}{u}(\alpha )-\underline{u}(\alpha )\right)+\underline{u}(\alpha )$, where *α* and *β* ∈ [0,1].

**Definition 5 (fuzzy arithmetic)**

For any two arbitrary fuzzy numbers $\tilde{x}=[\underline{x}(\alpha ),\overset{-}{x}(\alpha )]$, $\tilde{y}=[\underline{y}\left(\alpha \right),\overset{-}{y}\left(\alpha \right)]$, and scalar *k*, fuzzy arithmetics are defined as follows:

- $\tilde{x}=\tilde{y}$ if and only if $\underline{x}(\alpha )=\underline{y}(\alpha )$ and $\bar{x}(\alpha )=\bar{y}(\alpha )$,
- $\tilde{x}+\tilde{y}=\left[\underline{x}\left(\alpha \right)+\underline{y}\left(\alpha \right),\bar{x}+\bar{y}(\alpha )\right]$,
- $\tilde{x}\times \tilde{y}=\left[\begin{array}{c} \min\left(\underline{x}(\alpha )\times \underline{y}(\alpha ),\underline{x}(\alpha )\times \overset{-}{y}(\alpha ),\overset{-}{x}(\alpha )\times \underline{y}(\alpha ),\overset{-}{x}(\alpha )\times \overset{-}{y}(\alpha )\right),\\ \max\left(\underline{x}(\alpha )\times \underline{y}(\alpha ),\underline{x}(\alpha )\times \overset{-}{y}(\alpha ),\overset{-}{x}(\alpha )\times \underline{y}(\alpha ),\overset{-}{x}(\alpha )\times \overset{-}{y}(\alpha )\right) \end{array}\right]$,
- $k\tilde{x}=\{\begin{array}{cc} {}[k\overset{-}{x}(\alpha ),k\underline{x}(\alpha )], & k<0\\ {}[k\underline{x}(\alpha ),k\overset{-}{x}(\alpha )], & k\geq 0 \end{array}$.

## 3. Double Parametric Based Fuzzy Vibration Equation 

Here, we first convert the fuzzy vibration differential equation to interval based fuzzy differential equation using single parametric form. Then by using double parametric form, interval based fuzzy differential equation is reduced to crisp vibration equation. Now, we apply ADM to solve the corresponding differential equation. Let us now consider the fuzzy vibration equation

(6) ∂2v~∂r2+1r∂v~∂r=1c~2∂2v~∂t2, r≥0,  t≥0.

The above equation may be written as

(7) ∂2v~∂t2=c~2(∂2v~∂r2+1r∂v~∂r),

with fuzzy initial conditions

(8) v~(r,0)=(0.8,1,1.2)f(r),

(9) v~′(r,0)=c~g(r),

where $\tilde{v}(r,t)$ represents the uncertain displacement and $\tilde{c}$ is the wave velocity of free vibration.

We may consider (7) as

(10) Lttv~(r,t)=c~2(Lrrv~(r,t)+1rLrv~(r,t)),

where *L*_*tt*_ ≡ ∂^2^/∂*t* ^2^, *L*_*rr*_ ≡ ∂^2^/∂*r* ^2^, and *L*_*r*_ ≡ ∂/∂*r*.

As per single parametric form we may write the above fuzzy vibration equation (10) as

(11) Lttv~(r,t;α)=[Lttv_(r,t;α),Lttv−(r,t;α)]=[c_(α),c−(α)]2([Lrrv_(r,t),Lrrv−(r,t)]+1r[Lrv_(r,t),Lrv−(r,t)])

subject to fuzzy initial condition

(12) [v_(r,0;α),v−(r,0;α)]=[0.2α+0.8,1.2−0.2α]f(r),

(13) [v_′(r,0;α),v−′(r,0;α)]=[c_(α),c−(α)]g(r),where  α∈[0,1].

One may see here that (11) with the fuzzy initial conditions is all in interval form. Next using double parametric form (as discussed in Definition 4), (11) can be expressed as

(14) {β(Lttv−(r,t;α)−Lttv_(r,t;α))+Lttv_(r,t;α)}  ={β(c−(α)−c_(α))+c_(α)}2({β(Lrrv_(r,t;α)−Lrrv−(r,t;α))+Lrrv_(r,t;α)}+1r{β(Lrv_(r,t;α)−Lrv−(r,t;α))+Lrv_(r,t;α)})

subject to the initial conditions

(15) {β(v_(r,0;α)−v−(r,0;α))+v_(r,0;α)}  ={β(0.4−0.4α)+(0.2α+0.8)}f(r),{β(v_′(r,0;α)−v−′(r,0;α))+v_′(r,0;α)}  ={β(c−(α)−c_(α))+c_(α)}g(r),where,α,β∈[0,1].

It is now worth mentioning that (14) with the interval initial conditions is all now converted to crisp form in terms of *α* and *β*.

Let us now denote

(16) {β(Lttv−(r,t;α)−Lttv_(r,t;α))+Lttv_(r,t;α)}  =Lttv~(r,t;α,β),{β(Lrrv_(r,t;α)−Lrrv−(r,t;α))+Lrrv_(r,t;α)}  =Lrrv~(r,t;α,β),{β(Lrv_(r,t;α)−Lrv−(r,t;α))+Lrv_(r,t;α)}  =Lrv~(r,t;α,β),{β(c−(α)−c_(α))+c_(α)}=c~(α,β),{β(v_(r,0;α)−v−(r,0;α))+v_(r,0;α)}=v~(r,0;α,β),{β(v_′(r,0;α)−v−′(r,0;α))+v_′(r,0;α)}=v~′(0;α,β).

Substituting these values in (14) we get

(17) Lttv~(r,t;α,β)=(c~(α,β))2 ×(Lrrv~(r,t;α,β)+1rLrv~(r,t;α,β)),

with initial conditions

(18) v~(r,0;α,β)={β(0.4−0.4α)+(0.2α+0.8)}f(r),v~′(r,0;α,β)={β(c−(α)−c_(α))+c_(α)}g(r).

Solving the corresponding crisp differential equation one may get the solution as $\tilde{v}(r,t;\alpha ,\beta )$. To obtain the lower and upper bound of the solution in single parametric form we may put *β* = 0 and 1, respectively. This may be represented as $\tilde{v}(r,t;\alpha ,0)=\underline{v}(r,t,\alpha )$ and $\tilde{v}(r,t,\alpha ,1)=\overset{-}{v}(r,t,\alpha )$. Similarly other results may be obtained by plugging in different values of *α* and *β*.

### 3.1. Application of ADM [30, 31] to Uncertain Vibration Equation of Large Membranes with the Proposed Methodology

We have applied Adomian decomposition method to solve (17) and applying the operator *L*_*tt*_^−1^ (which is the inverse operator of *L*_*tt*_) on both sides of (17), the equivalent expression is

(19) v~(r,t;α,β)  =v~(r,0;α,β)   +tv~t(r,0;α,β)+(c~(α,β))2   ×(Ltt−1Lrrv~(r,t;α,β)+1rLtt−1Lrv~(r,t;α,β)),

where

(20) Ltt−1Lttv~(r,t;α,β)=v~(r,t;α,β) −v~(r,0;α,β)−tv~t(r,0;α,β).

According to Adomian decomposition [30, 31] we assume an infinite series solution for unknown function *v*(*r*, *t*; *α*, *β*) as

(21) v~(r,t;α,β)=∑n=0∞v~n(r,t;α,β),

where the components ${\tilde{v}}_{0}(r,t;\alpha ,\beta ),{\tilde{v}}_{1}(r,t;\alpha ,\beta ),{\tilde{v}}_{2}(r,t;\alpha ,\beta ),\ldots$ are usually determined by

(22) v~0(r,t;α,β)=v~(r,0;α,β)+tv~t(r,0;α,β),v~1(r,t;α,β)=Ltt−1((c~(α,β))2Lrrv~0(r,t;α,β)+(c~(α,β))2rLrv~0(r,t;α,β)),v~2(r,t;α,β)=Ltt−1((c~(α,β))2Lrrv~1(r,t;α,β)+(c~(α,β))2rLrv~1(r,t;α,β)),v~3(r,t;α,β)=Ltt−1((c~(α,β))2Lrrv~2(r,t;α,β)+(c~(α,β))2rLrv~2(r,t;α,β)),⋮

and so on.

Now substituting the above terms in (21) one may get the approximate solution of (17) as follows:

(23) v~(r,t;α,β)=v~0(r,t;α,β)+v~1(r,t;α,β) +v~2(r,t;α,β)+v~3(r,t;α,β)+⋯.

The above series converge very rapidly [37–39] and the rapid convergence means that only few terms are required to get the approximate solutions.

## 4. Solution Bounds for Particular Cases

In this section we have considered fuzzy initial conditions in single parametric form as $\tilde{v}(r,0;\alpha )=\left\lfloor 1-0.1\sqrt{-2\log_{e}\alpha },1+0.1\sqrt{-2\log_{e}\alpha }\right\rfloor f(r)$, $\tilde{\dot{v}}\left(r,0;\alpha \right)=\lfloor 6-0.1\sqrt{-2\log_{e}\alpha },6$  +  $0.1\sqrt{-2\log_{e}\alpha }\rfloor g(r)$ and the wave velocity as $\tilde{c}=\left\lfloor 6-0.1\sqrt{-2\log_{e}\alpha },6+0.1\sqrt{-2\log_{e}\alpha }\right\rfloor$. Depending upon the functions *f*(*r*) and *g*(*r*) we will have different cases [1] which are discussed in the following paragraphs for finding the uncertain solution bounds.

**Case 1**

Here we have taken*f*(*r*) = *r* ^2^ and *g*(*r*) = *r* in the above fuzzy initial conditions. Hence (10) will become

(24) Lttv~(r,t)=[6−0.1−2log⁡e⁡α,6+0.1−2log⁡e⁡α]2 ×(Lrrv~(r,t)+1rLrv~(r,t)).

Using double parametric form, (17) and the corresponding fuzzy initial conditions will become

(25) Lttv~(r,t;α,β)  =(β(0.2−2log⁡e⁡α)   +(6−0.1−2log⁡e⁡α))2   ×(Lrrv~(r,t;α,β)+1rLrv~(r,t;α,β)),

(26) v~(r,0;α,β)=β(0.2−2log⁡e⁡α) +(1−0.1−2log⁡e⁡α)f(r),

(27) v˙~(r,0;α,β)=β(0.2−2log⁡e⁡α) +(6−0.1−2log⁡e⁡α)g(r).

Let us now denote

(28) β(0.2−2log⁡e⁡α)+(1−0.1−2log⁡e⁡α)=ηβ(0.2−2log⁡e⁡α)+(6−0.1−2log⁡e⁡α)=δ.

Applying ADM we have

(29) v~0(r,t;α,β)=δrt+ηr2,v~1(r,t;α,β)=2ηδ2t2+δ3t36r,v~2(r,t;α,β)=δ5t5120r3,v~3(r,t;α,β)=δ7t7560r5,

and so on.

In the similar manner, higher order approximation may be obtained as discussed above.

Therefore, the solution can be written as

(30) v~(r,t;α,β)=r2(η+δ(tr)+2ηδ2(tr)2+δ36(tr)3+δ5120(tr)5+δ7560(tr)7+⋯).

To obtain the solution bounds in single parametric form we may put *β* = 0 and 1 in (30) for lower and upper bounds of the solution, respectively. So we get

(31) v_(r,t;α,0)  =r2((1−0.1−2log⁡e⁡α)     +(6−0.1−2log⁡e⁡α)(tr)     +2(1−0.1−2log⁡e⁡α)     ×(6−0.1−2log⁡e⁡α)2(tr)2     +(6−0.1−2log⁡e⁡α)36(tr)3     +(6−0.1−2log⁡e⁡α)5120(tr)5     +(6−0.1−2log⁡e⁡α)7560(tr)7+⋯),v−(r,t;α,1)  =r2((1+0.1−2log⁡e⁡α)     +(6+0.1−2log⁡e⁡α)(tr)     +2(1+0.1−2log⁡e⁡α)     ×(6+0.1−2log⁡e⁡α)2(tr)2     +(6+0.1−2log⁡e⁡α)36(tr)3     +(6+0.1−2log⁡e⁡α)5120(tr)5     +(6+0.1−2log⁡e⁡α)7560(tr)7+⋯).

One may note that in the special case when *α* = 1 and wave velocity *c* = 6, the crisp results obtained by the proposed method are exactly the same as that of the solution obtained by Yildirim et al. [1]. The above series will be convergent for the values of |*t*/*r*| ≤ 1, that is, for large membrane and small range of time.

**Case 2**

Now we consider *f*(*r*) = *r* and *g*(*r*) = 1.

Again, by applying the procedure discussed previously, we get the solution

(32) v~0(r,t;α,β)=δt+ηr,v~1(r,t;α,β)=δ2ηt22r,v~2(r,t;α,β)=δ4ηt424r3,v~3(r,t;α,β)=ηδ6t680r5,

and so on.

The solution in general form may be obtained as

(33) v~(r,t;α,β)=r(δ(tr)+η+δ2η2(tr)2  +δ4η24(tr)4+ηδ680(tr)6+⋯).

Putting *β* = 0 and 1 in $\tilde{v}\left(r,t;\alpha ,\beta \right)$ we get the lower and upper bounds of the fuzzy solutions, respectively, as

(34) v_(r,t;α,0)  =r((1−0.1−2log⁡e⁡α)+(6−0.1−2log⁡e⁡α)(tr)+(6−0.1−2log⁡e⁡α)2(1−0.1−2log⁡e⁡α)2(tr)2+(6−0.1−2log⁡e⁡α)4(1−0.1−2log⁡e⁡α)24(tr)4+(1−0.1−2log⁡e⁡α)(6−0.1−2log⁡e⁡α)680×(tr)6+⋯),v−(r,t;α,1)  =r((1+0.1−2log⁡e⁡α)+(6+0.1−2log⁡e⁡α)(tr)+(6+0.1−2log⁡e⁡α)2(1+0.1−2log⁡e⁡α)2(tr)2+(6+0.1−2log⁡e⁡α)4(1−0.1−2log⁡e⁡α)24(tr)4+(1−0.1−2log⁡e⁡α)(6+0.1−2log⁡e⁡α)680×(tr)6+⋯).

Solution obtained by proposed method for *α* = 1 and the wave velocity *c* = 6 is again found to be exactly the same as that of (crisp result) Yildirium et al. [1].

**Case 3**

Next we take $f(r)=\sqrt{r}$ and $g(r)=1/\sqrt{r}$.

By following the proposed method with ADM, we get the solution in double parametric form as

(35) v~(r,t;α,β)=r(η+δ(tr)+δ2η8(tr)2+δ324(tr)3+3δ4η128(tr)4+δ5384(tr)5+49δ6η5120(tr)6+9δ77168(tr)7⋯).

The lower and upper bounds of the fuzzy solutions may again be written as

(36) v_(r,t;α,0)  =r((1+0.1−2log⁡e⁡α)+(6+0.1−2log⁡e⁡α)(tr)+(6+0.1−2log⁡e⁡α)2(1+0.1−2log⁡e⁡α)8×(tr)2+(6+0.1−2log⁡e⁡α)324(tr)3+3(6−0.1−2log⁡e⁡α)4(1−0.1−2log⁡e⁡α)128×(tr)4+(6−0.1−2log⁡e⁡α)5384(tr)5+49(6−0.1−2log⁡e⁡α)6(1−0.1−2log⁡e⁡α)5120×(tr)6+9(6−0.1−2log⁡e⁡α)77168(tr)7⋯),v−(r,t;α,1)  =r((1+0.1−2log⁡e⁡α)+(6+0.1−2log⁡e⁡α)(tr)+(6+0.1−2log⁡e⁡α)2(1+0.1−2log⁡e⁡α)8×(tr)2+(6+0.1−2log⁡e⁡α)324(tr)3+3(6+0.1−2log⁡e⁡α)4(1−0.1−2log⁡e⁡α)128×(tr)4+(6+0.1−2log⁡e⁡α)5384(tr)5+49(6+0.1−2log⁡e⁡α)6(1−0.1−2log⁡e⁡α)5120×(tr)6+9(6+0.1−2log⁡e⁡α)77168(tr)7⋯).

**Case 4**

Consider

(37) f(r)=r2,g(r)=1.

In this case we have

(38) v~0(r,t;α,β)=δt+ηr2,v~1(r,t;α,β)=2δ2ηt2,v~2(r,t;α,β)=0,v~n(r,t;α,β)=0, for  n≥2.  

Therefore the solution in double parametric form is as follows:

(39) v~(r,t;α,β)=ηr2+δt+2δ2ηt2.

The lower and upper bounds of the fuzzy solution are obtained as

(40) v_(r,t;α,0)=(1−0.1−2log⁡e⁡α)r2+(6−0.1−2log⁡e⁡α)t +2(6−0.1−2log⁡e⁡α)2(1−0.1−2log⁡e⁡α)t2,v−(r,t;α,1)=(1+0.1−2log⁡e⁡α)r2+(6+0.1−2log⁡e⁡α)t+2(6+0.1−2log⁡e⁡α)2(1+0.1−2log⁡e⁡α)t2.

Again one may see that the solution obtained by proposed method for *α* = 1 and the wave velocity *c* = 6 exactly agrees with the solution of Yildirium et al. [1].

**Case 5**

Finally we have considered *f*(*r*) = *r* ^2^ and *g*(*r*) = *r* ^2^.

We have the solutions in this case as

(41) v~0(r,t;α,β)=δr2t+ηr2,v~1(r,t;α,β)=23δ3t3+2δ2ηt2,v~2(r,t;α,β)=0,v~n(r,t;α,β)=0,   for  n≥2

and finally one may write

(42) v~(r,t;α,β)=ηr2+δr2t+23δ3t3+2δ2ηt2.

Lower and upper bounds of the solutions, respectively, are

(43) v_(r,t;α,0)=(1−0.1−2log⁡e⁡α)r2 +(6−0.1−2log⁡e⁡α)r2t +23(6−0.1−2log⁡e⁡α)3t3 +2(6−0.1−2log⁡e⁡α)2  ×(1−0.1−2log⁡e⁡α)t2,v−(r,t;α,1)=(1+0.1−2log⁡e⁡α)r2+(6+0.1−2log⁡e⁡α)r2t+23(6+0.1−2log⁡e⁡α)3t3+2(6−0.1−2log⁡e⁡α)2×(1+0.1−2log⁡e⁡α)t2.

## 5. Numerical Results and Discussions

In this section, we present numerical solution of uncertain vibration equation for large membranes using ADM. It is a gigantic task to include here all the results with respect to various parameters and initial conditions involved in the corresponding fuzzy differential equation. So, some particular values of the parameters are taken to compute the results with the above cases. Obtained results by the present analysis are compared with the existing solution of [1] in special cases to show the validation of the proposed analysis. Computed results are depicted in term of plots.

**Figure 1:**
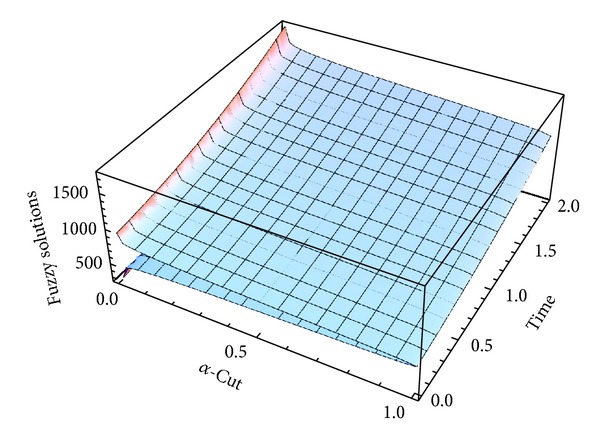
Fuzzy displacement at *r* = 25 of Case 1.

**Figure 2:**
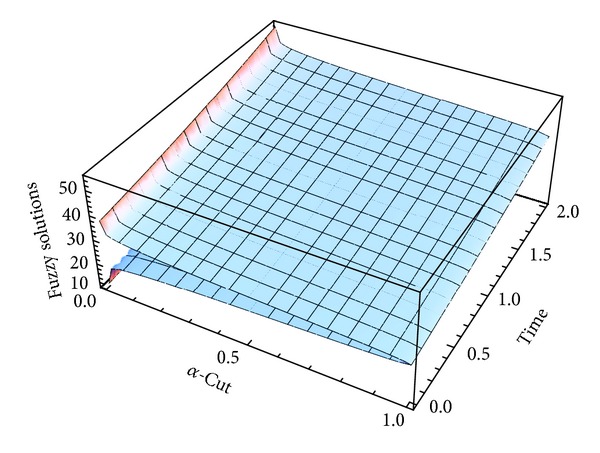
Fuzzy solution at *r* = 25 of Case 2.

**Figure 3:**
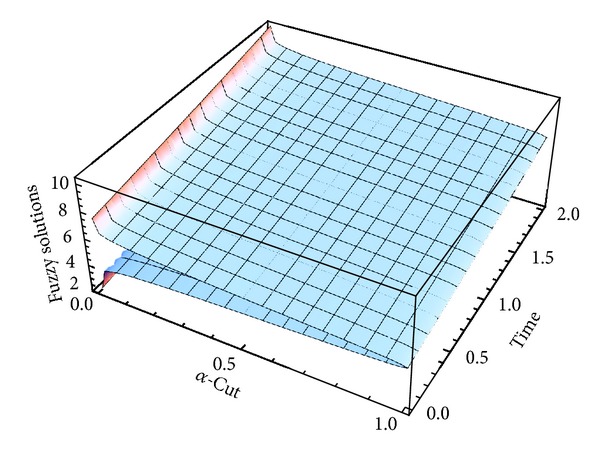
Fuzzy solution at *r* = 25 of Case 3.

**Figure 4:**
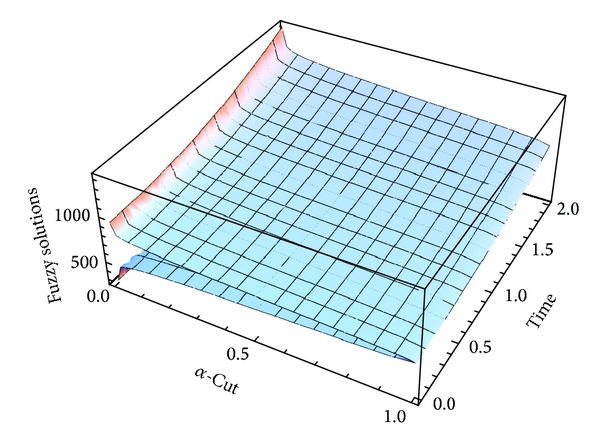
Fuzzy solution at *r* = 25 of Case 4.

**Figure 5:**
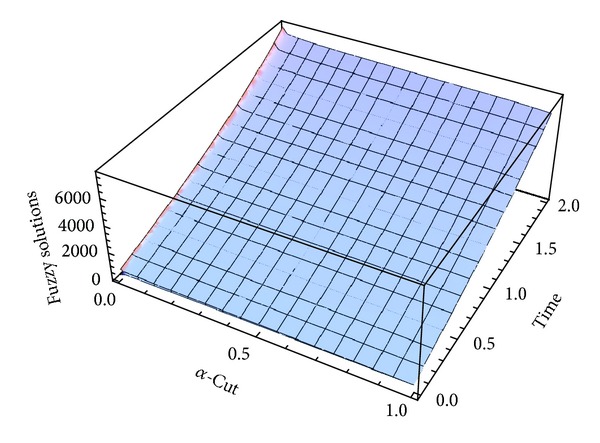
Fuzzy solution at *r* = 25 of Case 5.

**Figure 6:**
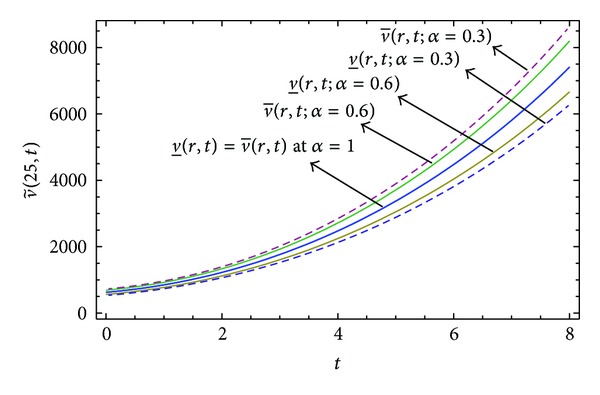
Interval solution of Case 1.

**Figure 7:**
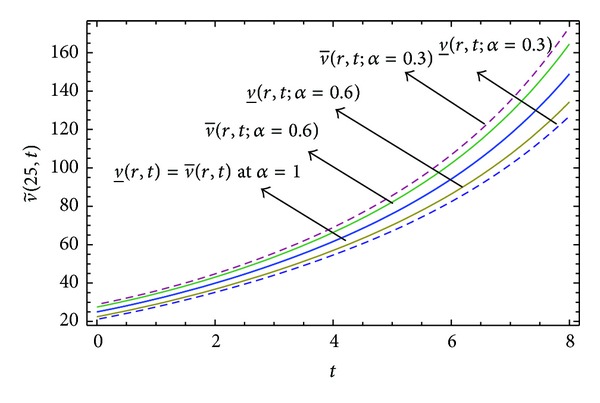
Interval solution of Case 2.

**Figure 8:**
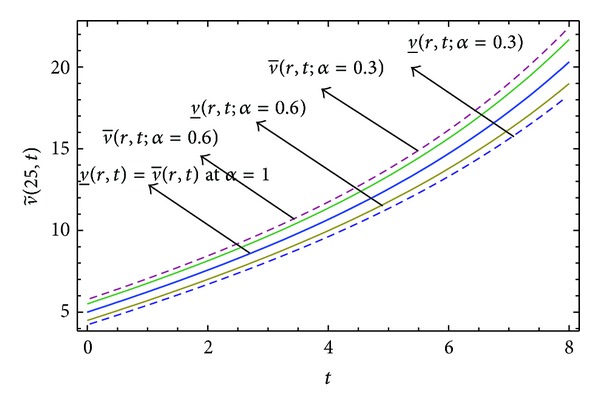
Interval solution of Case 3.

**Figure 9:**
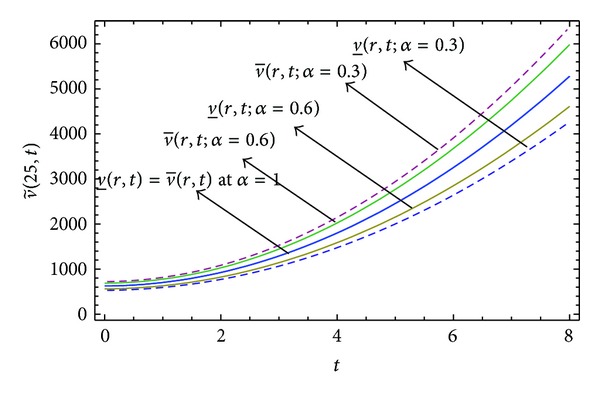
Interval solution of Case 4.

**Figure 10:**
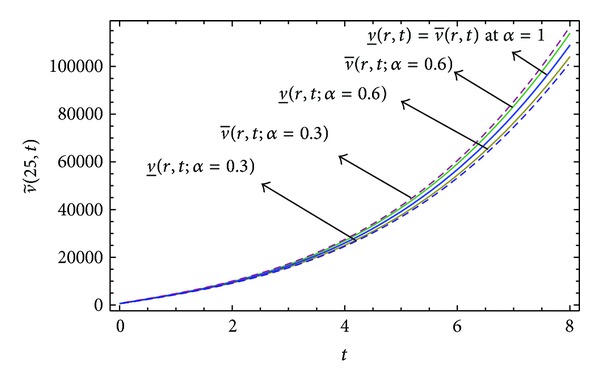
Interval solution of Case 5.

**Figure 11:**
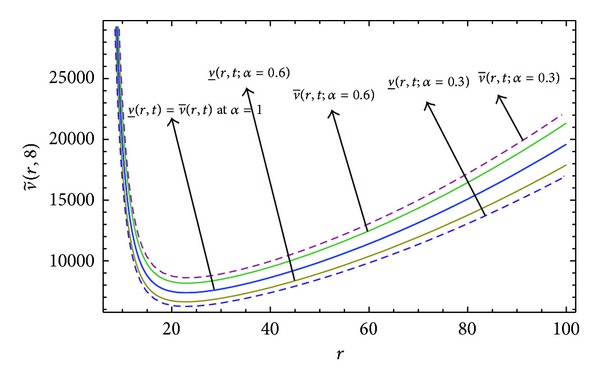
Interval solution of Case 1 at *t* = 8.

**Figure 12:**
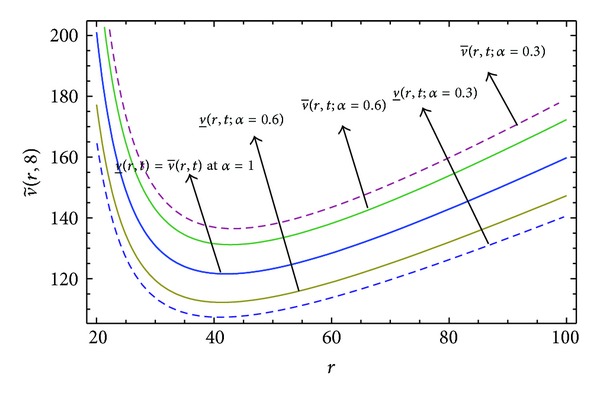
Interval solution of Case 2 at *t* = 8.

**Figure 13:**
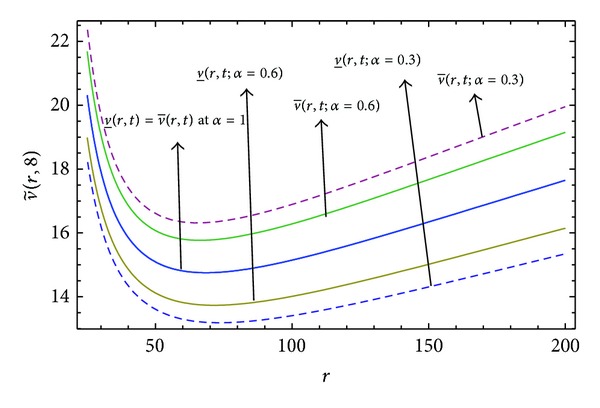
Interval solution of Case 3 at *t* = 8.

Here numerical computations have been done by truncating the infinite series (31), (34), (36), (40), and (43) to a finite number (*n* = 3) of terms. Gaussian fuzzy solution for particular Cases 1, 2, 3, 4, and 5 are depicted in Figures 1, 2, 3, 4, and 5 by varying time *t* from 0 to 2 and for a particular value of radius of membrane *r* = 25. Next, interval solutions for *α*-cut 0.3, 0.6, and 1 and varying *t* from 0 to 8 for different cases have been given in Figures 6, 7, 8, 9, and 10 respectively, with radius of membrane, *r* = 25. One may see from these figures that the crisp result (*α* = 1) is the central line and the interval solutions are spread both sides of the crisp results. Similarly for *t* = 8 and different values of *r* (for all the five cases) we plot the interval solutions in Figures 11, 12, 13, and 15. It may be worth mentioning that for all the cases present results with *α* = 1 exactly agree with the solution of [1].

**Figure 14:**
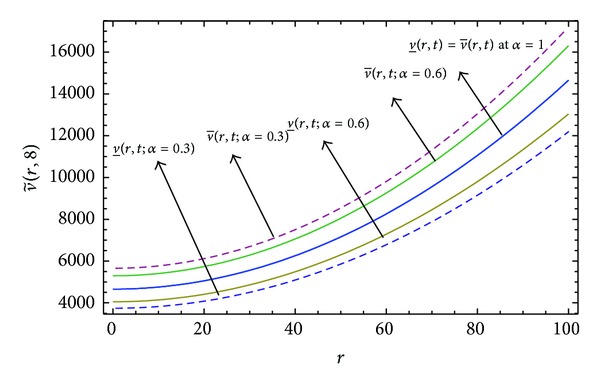
Interval solution of Case 4 at *t* = 8.

**Figure 15:**
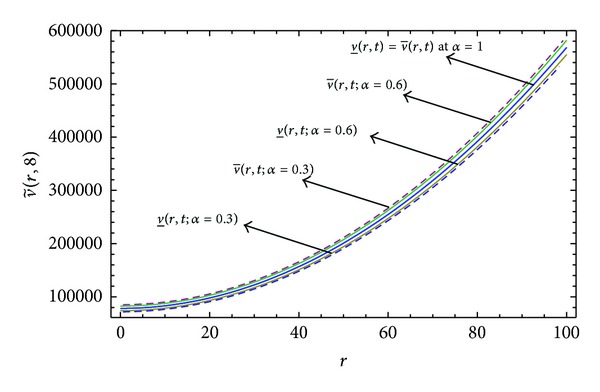
Interval solution of Case 5 at *t* = 8.

Also it is interesting to note from Figures 6 to 10 that the left and right bounds of the uncertain displacement, that is, $\tilde{v}(r,t)$ (with particular values of *α* and *r*), gradually increase with increase in time. And in Figures 11 to 13 for particular value of *α* and *t*, the uncertain displacement first decreases and then increases with increase in radius of membrane *r* for Cases 1 to 3. But Figures 14 and 15 for Cases 4 and 5 show that $\tilde{v}(r,t)$ increases with increase in *r*. The rate of increase in uncertain displacement is faster in Case 1 than in Cases 2 and 3. Rate of increase in uncertain displacement in Case 5 is faster than that in Case 4.

## 6. Conclusions

In this paper double parametric form of fuzzy numbers has successfully been applied to the solution of fuzzy vibration equation for large membranes using ADM. The double parametric form approach is found to be easy and straight forward. Here, performance of the method is shown by using Gaussian fuzzy number. It is interesting to note for *α* = 1 the lower bound solution is equal to the upper bound solution. Though the solution ADM is of the form of an infinite series, it can be written in a closed form. The main advantage of ADM is the capability to achieve exact solution and rapid convergence with few terms.
